# Usefulness of Texture Analysis Using MRI in Thymic Epithelial Tumors

**DOI:** 10.7759/cureus.111959

**Published:** 2026-07-02

**Authors:** Xiao-Feng Li, Kazuhiro Saito, Shuhei Shibukawa, Daisuke Yoshimaru, Yoichi Araki, Yuki Takara, Jinho Partk, Jun Matubayashi, Norihiko Ikeda, Masanori Ishida

**Affiliations:** 1 Radiology, Xuzhou Cancer Hospital, Xuzhou, CHN; 2 Radiology, Tokyo Medical University, Tokyo, JPN; 3 Anatomic Pathology, Tokyo Medical University, Tokyo, JPN; 4 Thoracic Surgery, Tokyo Medical University, Tokyo, JPN

**Keywords:** mediastinum, mri, texture analysis, thymic epithelial tumor, who classification

## Abstract

Background

MRI radiomics has recently gained attention for evaluating intratumoral heterogeneity in thoracic malignancies. Thymic epithelial tumors (TETs) include a wide spectrum from the low-risk thymoma group (LRG) to thymic carcinoma (TC), but imaging-based differentiation remains challenging. This study aimed to assess the utility of MRI texture analysis for differentiating TET subtypes according to the 2021 WHO classification.

Methods

Forty-seven patients with histologically proven TETs (19 LRG, 18 high-risk thymoma group (HRG), and 10 TC) underwent preoperative MRI including T1-, T2-, diffusion-weighted (DWI), and apparent diffusion coefficient (ADC) sequences. Texture analysis was performed using LIFEx software (version 7.6.0; French Alternative Energies and Atomic Energy Commission, Paris, France). A total of 41 texture features were extracted from manually delineated three-dimensional tumor volumes. Statistical comparisons were performed using Kruskal-Wallis and Mann-Whitney U tests with Benjamini-Hochberg false discovery rate correction. Diagnostic performance was evaluated using receiver operating characteristic (ROC) analysis.

Results

Among the 47 lesions, 24, 28, and 37 texture parameters differed significantly between LRG-HRG, LRG-TC, and HRG-TC, respectively. The highest area under the curve (AUC) values were 0.785 for DWI coefficient of variation in differentiating LRG from HRG, 0.933 for T1WI morphological radius in differentiating LRG from TC, and 0.867 for ADC intensity variance in differentiating HRG from TC.

Conclusions

MRI-based texture analysis provides a promising noninvasive approach for differentiating thymic epithelial tumor subtypes according to the WHO classification. Sequence-specific texture parameters demonstrated moderate to high diagnostic performance, particularly for differentiating low-risk thymoma from thymic carcinoma. These findings suggest that MRI radiomics may contribute to preoperative risk stratification and treatment planning in patients with thymic epithelial tumors.

## Introduction

Thymic epithelial tumors are classified into thymomas (types A, AB, B1, B2, and B3) and thymic carcinomas according to the 2021 World Health Organization (WHO) classification [1]. The histological classification of thymic tumors is strongly correlated with prognosis, with reported five-year survival rates of 55% for thymic carcinoma and 90% for thymomas [2-4]. It has been reported that the WHO histological type significantly influences the recurrence rate in lower stages, such as stages Ⅰ and Ⅱ [5]. Therefore, establishing a histological tumor diagnosis before treatment is valuable for determining the treatment strategy. Several studies have evaluated the relationship between radiological examinations and WHO classification. Tomiyama et al. reported that on computed tomography (CT), thymomas of types A and B1 typically present as round, homogeneous solid tumors with smooth margins, whereas types B2, B3, and thymic carcinoma exhibit lobulated morphology, irregular margins, and heterogeneous internal structures [6]. Positron emission tomography (PET) is useful for detecting metastatic lesions; however, its ability to distinguish histological differences is limited [7].

Conversely, magnetic resonance imaging (MRI) has been reported to be superior to CT in differentiating thymic hyperplasia and thymic cysts from thymic epithelial tumors [8], and several studies have indicated the utility of MRI in diagnosing thymic carcinoma. Several studies suggest thymic carcinoma in cases of low apparent diffusion coefficient (ADC) values [9-11]. However, conventional MRI features often overlap among WHO subtypes. Radiomics and texture analysis have emerged as quantitative approaches to characterize intratumoral heterogeneity beyond visual assessment. Previous CT-based studies have shown the potential of radiomic signatures in differentiating thymoma risk categories. However, MRI texture analysis of TETs remains underexplored. Li et al. demonstrated that combining ADC and diffusion-weighted (DWI)-derived texture features improved subtype differentiation, emphasizing multiparametric MRI’s potential [12].

Therefore, we investigated whether MRI-derived texture analysis could differentiate thymic epithelial tumor subtypes according to the 2021 WHO classification. The primary objective of this study was to determine whether MRI-derived texture features obtained from multiple MRI sequences could differentiate low-risk thymoma, high-risk thymoma, and thymic carcinoma according to the 2021 WHO classification. A secondary objective was to identify the most diagnostically useful sequence-specific texture features for each comparison and to explore their potential clinical value for preoperative risk stratification.

## Materials and methods

Participants

**Figure 1:**
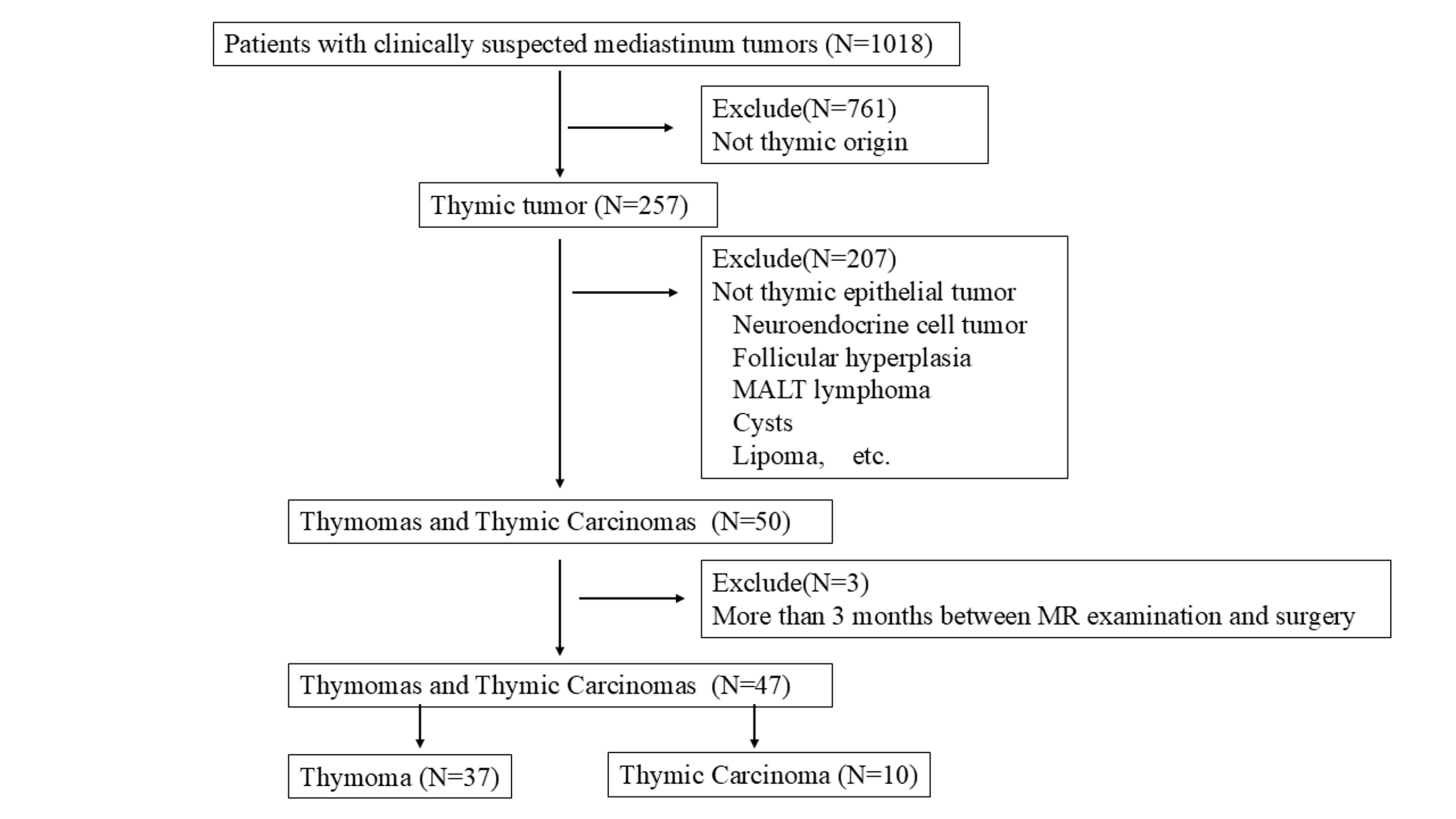
Flowchart illustrating the case selection process in this study

This retrospective study was approved by the Tokyo Medical University Institutional Review Board (approval no. T2024-0083), and the requirement for informed consent was waived with opt-out notification. Patients who underwent surgical treatment for mediastinal tumors at our institution between December 2007 and May 2024 were retrospectively reviewed. The inclusion criteria were: (1) Histological evaluation available based on the 2021 WHO classification of thymic and mediastinal tumors [1]; (2) The availability of MRI images, including T1-weighted, T2-weighted, and diffusion-weighted images (DWI); (3) No chemotherapy was administered before the MRI. The exclusion criteria were: (1) Histological diagnosis other than thymoma or thymic carcinoma; (2) An interval of more than three months between MRI and surgery; (3) Tumor size ˂ 64 pixels. The flow chart of case selection in this study is shown in Figure 1.

MRI acquisition

MRI was performed using a 3-Tesla system (Skyra, Vida, Siemens Medical System, Erlangen, Germany). The imaging sequences are as follows: T1-weighted images (T1WI) were acquired using a three-dimensional Dixon volumetric interpolated breath-hold examination (3D-VIBE Dixon), capturing both in-phase and opposed-phase images. The sequence parameters were as follows: TR/TE = 6.7/2.39, 4.77 ms; field of view = 320 × 400 mm²; voxel size = 0.6 × 0.6 × 2 mm³; slice thickness = 2 mm; slices per slab = 72; flip angle = 15°; parallel acquisition technique (PAT) factor = 2; concatenations = 1; bandwidth = 630 Hz/pixel; scanning time = 21 seconds. Axial T2-weighted images (T2WI) were acquired using a turbo spin echo sequence. The sequence parameters were as follows: Repetition Time/Echo Time (TR/TE) = 4000/83 ms; field of view = 300 × 400 mm²; voxel size = 0.6 × 0.6 × 5 mm³; slice thickness = 5 mm; gap = 0.5 mm; flip angle = 130°; number of slices = 20; PAT factor = 2; concatenations = 2; bandwidth = 300 Hz/pixel; scanning time = 46 seconds. DWI was acquired using a single-shot spin-echo planar imaging sequence. The sequence parameters were as follows: TR/TE = 5200/67 ms; field of view = 267 × 400 mm²; voxel size = 1 × 1 × 5 mm³; slice thickness = 5 mm; gap = 0.5 mm; flip angle = 180°; number of slices = 20; PAT factor = 2; inversion time (TI) = 160 ms; concatenations = 2; bandwidth = 2368 Hz/pixel; b value = 0,800 s/mm²; scanning time = 1 minute 46 seconds.

Analysis

Initially, Digital Imaging and Communications in Medicine (DICOM) data of the eligible patients were obtained and converted to nii.gz format using MRIcroGL (version 1.2.20210317). Subsequently, the converted data were loaded into LIFEx (version 7.6.0; French Alternative Energies and Atomic Energy Commission, Paris, France). A board-certified radiologist with more than 17 years of experience manually delineated the volume of interest (VOI), excluding necrosis, calcification, and surrounding fat. The analysis was performed on T1-weighted images, T2-weighted images, diffusion-weighted images, and ADC maps. It should be noted that conventional mean ADC values were not evaluated. Instead, texture features were extracted from ADC maps in the same manner as for the other MRI sequences. The VOI was defined on each imaging slice and adjusted to encompass the entire tumor in three dimensions using coronal and sagittal sections. The texture analysis based on LIFEx features included the following: Intensity-based features, Intensity histogram features, Gray-Level Co-occurrence Matrix (GLCM) features, Neighborhood Gray-Level Difference Matrix (NGLDM) features, Gray-Level Run Length Matrix (GLRLM) features, and Gray-Level Zone Length Matrix (GLZLM) features.

Histological evaluation

Histopathological diagnosis was performed using surgical specimens by a board-certified pathologist with 30 years of experience. Thymic epithelial tumors were categorized into three groups based on the 2021 WHO classification [1]: 1. Low-risk group (LRG): thymoma types A, AB, and B1; 2. High-risk group (HRG): thymoma types B2 and B3; 3. Thymic carcinoma (TC).

Statistical analysis

**Figure 2:**
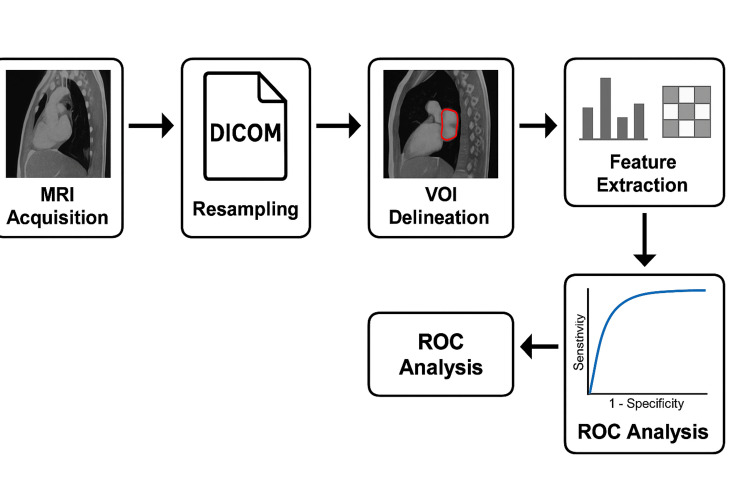
Workflow of this study Workflow for MRI texture analysis using LIFEx software (version 7.6.0; French Alternative Energies and Atomic Energy Commission, Paris, France), including Digital Imaging and Communications in Medicine (DICOM) import, preprocessing, volume of interest (VOI) delineation, feature extraction, and receiver operating characteristic (ROC) evaluation.

Continuous variables are presented as median (interquartile range), and categorical variables as counts and percentages. Normality testing was not performed due to the small sample size. Comparisons of texture features among the three groups (LRG, HRG, and TC) were performed using the Kruskal-Wallis test, followed by pairwise comparisons using the Mann-Whitney U test. To control for multiple comparisons arising from the large number of texture features, p-values were adjusted using the Benjamini-Hochberg false discovery rate (FDR) method. All extracted texture features were evaluated individually, and receiver operating characteristic (ROC) analysis was subsequently performed for texture features that showed statistically significant group differences after FDR correction. The area under the curve (AUC) with 95% confidence intervals (CI) was calculated using the DeLong method. All statistical analyses were conducted using R software (version 4.3.3; packages: pROC, stats) and Python (version 3.12). A two-sided p-value < 0.05 was considered statistically significant. The workflow of this study is shown in Figure 2.

## Results

A total of 47 cases comprising 47 lesions were examined. The ages ranged from 16 to 85 years, with a median age of 59 years. The participants included 22 males and 25 females. The distribution of lesions was as follows: 19 cases in the LRG of thymic epithelial tumors (type A: 1, type AB: 15, and type B1: 3), 18 cases in the HRG (type B2: 12, type B3: 6), and 10 cases of TC. The mean tumor size was 35.6 ± 44.5 cm^3^. The texture parameters that demonstrated significant differences between the LRG, HRG, and TC, along with the number of texture analyses performed for each sequence, are shown in Table 1.

**Table 1:** Number of texture parameters showing significant differences between low-risk, high-risk thymomas, and thymic carcinomas LRG: Low-risk thymoma group, HRG: High-risk thymoma group, TC: Thymic carcinoma, AUC: Area under the curve.

Comparison	Significant features (n)	Top feature	AUC (95% CI)	Adjusted p
LRG vs HRG	24	INTENSITY-BASED_IntensityBasedCoefficientOfVariation (DWI)	0.785 (0.65–0.90)	0.01
LRG vs TC	28	MORPHOLOGICAL_RadiusRoiNorm-MaxIntensityCoor-PerimeterCoor-3DSmallestDist (T1WI)	0.933 (0.85–0.99)	<0.001
HRG vs TC	37	INTENSITY-BASED_IntensityVariance (ADC)	0.867 (0.75–0.96)	<0.001

Twenty-four parameters exhibited significant differences between LRG and HRG, 28 between LRG and TC, and 37 between HRG and TC. No parameters showed significant differences across all three comparisons; however, 31 parameters showed significant differences in two of the three comparisons. Among these, only one parameter, GLZLM_LowGrayLevelZoneEmphasis derived from DWI, showed significant differences between LRG and HRG and between LRG and TC. Thirteen parameters showed significant differences between LRG and HRG, and between HRG and TC, while 17 parameters showed significant differences between LRG and TC, and between HRG and TC. The parameters that showed significant differences in only one combination were: 10 for LRG vs. HRG, 10 for LRG vs. TC, and 7 for HRG vs. TC. Detailed values of representative parameters and their diagnostic performance are summarized in the Appendix. The DWI coefficient of variation, T1 morphological radius, and ADC intensity variance showed the highest AUCs for differentiating LRG-HRG, LRG-TC, and HRG-TC, respectively.

**Figure 3:**
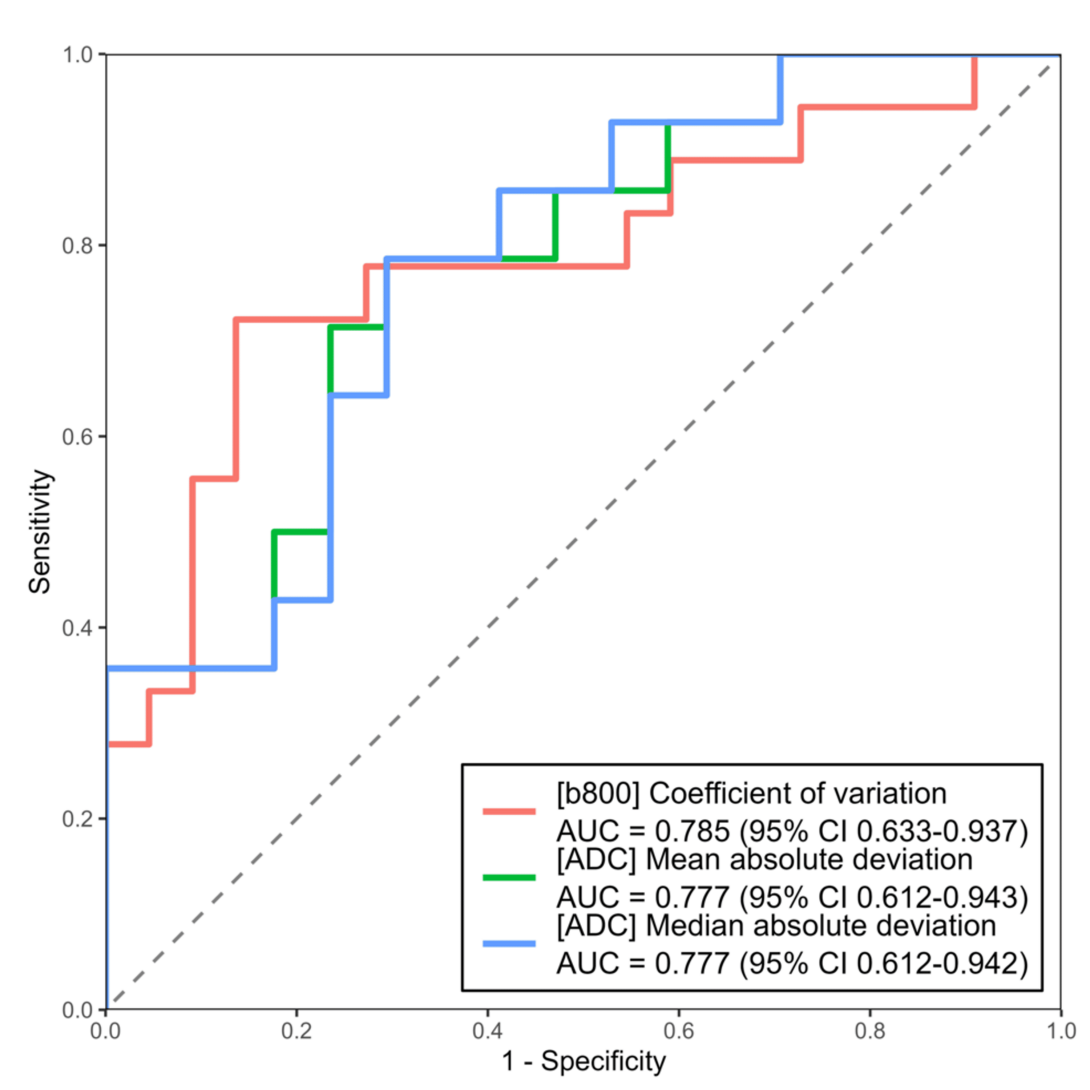
ROC curves for differentiating low-risk from high-risk thymoma The three top-performing individual features for the LRG-HRG comparison are shown. In this comparison, the highest-ranked features were mainly derived from diffusion-weighted imaging and ADC-related parameters. The legend lists the MRI sequence, feature name, area under the curve (AUC), and 95% confidence interval (CI) calculated by the DeLong method. ROC: Receiver operating characteristic, ADC: Apparent diffusion coefficient.

**Figure 4:**
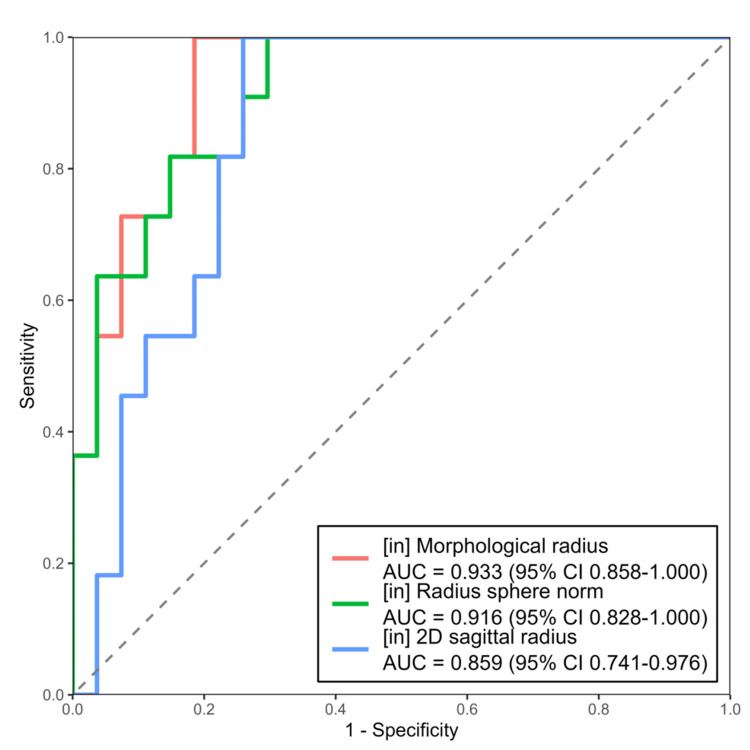
ROC curves for differentiating low-risk thymoma from thymic carcinoma The three top-performing individual features for the LRG-TC comparison are shown. The selected features were predominantly derived from in-phase T1-weighted imaging, reflecting the strong discriminatory performance of morphology-based parameters in this comparison. The legend lists the MRI sequence, feature name, area under the curve (AUC), and 95% confidence interval (CI) calculated by the DeLong method. ROC: Receiver operating characteristic, LRG: Low-risk thymoma group, TC: Thymic carcinoma.

**Figure 5:**
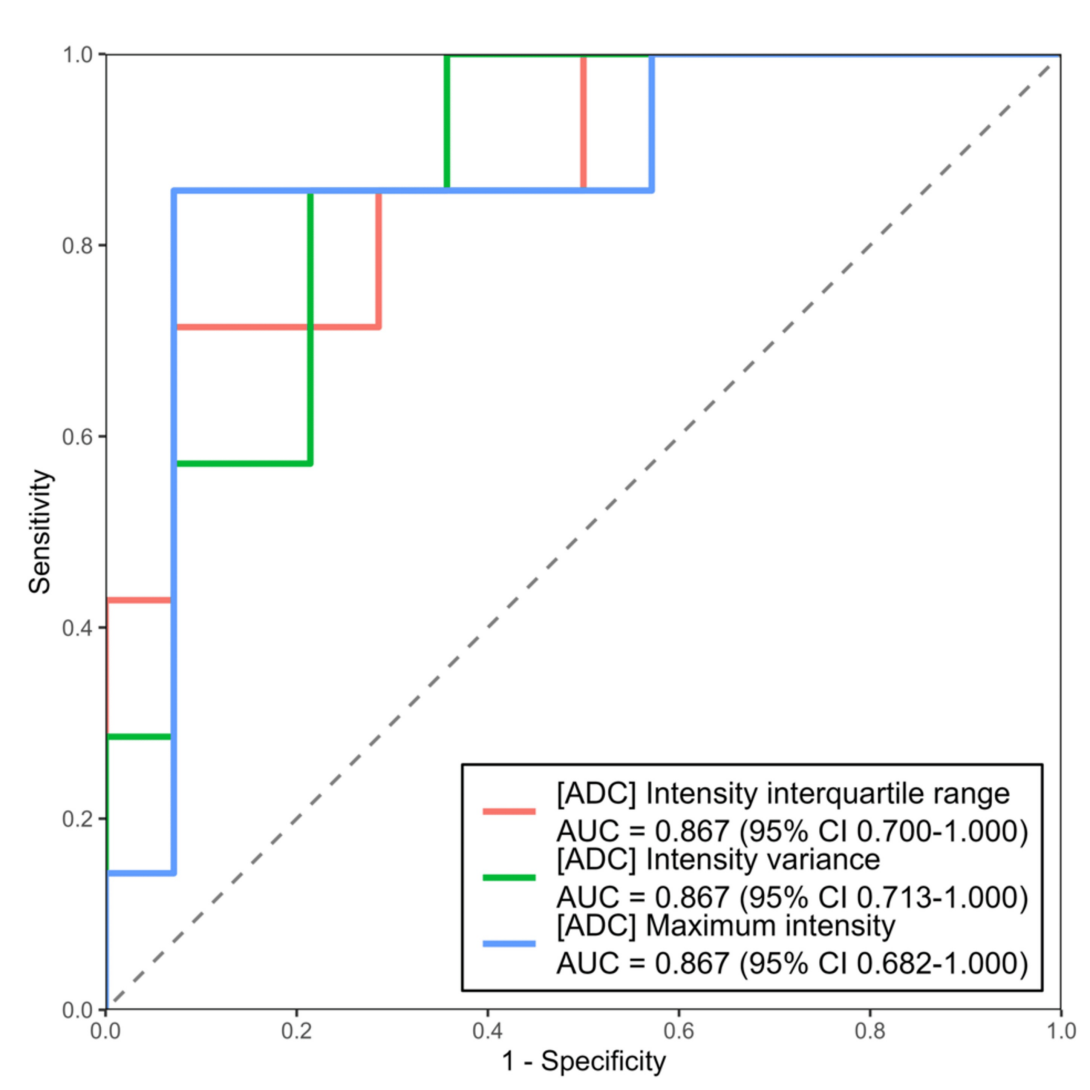
ROC curves for differentiating high-risk thymoma from thymic carcinoma The three top-performing individual features for the HRG-TC comparison are shown. In this comparison, the selected features were derived from ADC-based intensity parameters with similar diagnostic performance. The legend lists the MRI sequence, feature name, area under the curve (AUC), and 95% confidence interval (CI) calculated by the DeLong method. ROC: Receiver operating characteristic, ADC: Apparent diffusion coefficient, HRG: High-risk thymoma group, TC: Thymic carcinoma.

The ROC analysis for the differential diagnosis between LRG and HRG is shown in Figure 3. The highest area under the curve (AUC) was 0.785, observed for the INTENSITY-BASED_IntensityBasedCoefficientOfVariation derived from DWI. The ROC curve for differentiating LRG from TC is shown in Figure 4. The MORPHOLOGICAL_RadiusRoiNorm-MaxIntensityCoor-PerimeterCoor-3DSmallestDist, derived from the in-phase T1WI, yielded the highest AUC of 0.933. The ROC curve for the differentiation between HRG and TC is shown in Figure 5. The following four texture analyses derived from the ADC map demonstrated the highest AUC of 0.867: INTENSITY-BASED_IntensityVariance, INTENSITY-BASED_StandardDeviation, INTENSITY-BASED_MaximumIntensity, and INTENSITY-BASED_IntensityInterquartileRange.

The ROC curve for the differentiation between LRG and TC is shown in Figure 4. The MORPHOLOGICAL_RadiusRoiNorm-MaxIntensityCoor-PerimeterCoor-3DSmallestDist, derived from the in-phase T1WI, yielded the highest AUC of 0.933. The ROC curve for the differentiation between HRG and TC is shown in Figure 5. The following four texture analyses derived from the ADC map demonstrated the highest AUC of 0.867: INTENSITY-BASED_IntensityVariance, INTENSITY-BASED_StandardDeviation, INTENSITY-BASED_MaximumIntensity, and INTENSITY-BASED_IntensityInterquartileRange.

## Discussion

This study demonstrated the efficacy of MRI texture analysis in differentiating the malignancy of thymic epithelial tumors. DWI was instrumental in distinguishing between low-risk and high-risk tumors, with the coefficient of variation being particularly effective. In-phase T1WI was helpful in differentiating between low-risk tumors and carcinoma, particularly in identifying the location of the highest signal intensity. Both in-phase T1WI and the ADC map were effective in differentiating between high-risk tumors and TC, with the maximum value, variance, standard deviation, and interquartile range being particularly effective. However, no single texture feature demonstrated significance across all subgroup comparisons, possibly reflecting both the biological heterogeneity of thymic epithelial tumors and the exploratory nature of radiomic analysis. Texture analysis is a radiomic approach that quantitatively evaluates the spatial distribution and relationships of voxel intensities within a tumor, thereby providing information beyond conventional visual image assessment. These quantitative features are considered to reflect intratumoral heterogeneity, which is associated with variations in tumor cellularity, stromal composition, necrosis, hemorrhage, and other histopathological characteristics [13,14]. Consequently, MRI texture analysis has attracted increasing attention as a noninvasive imaging biomarker for tumor characterization, risk stratification, and treatment planning.

Significant differences were observed in the comparison between the LRG and the HRG and carcinoma only for the GLSZM_LargeZoneHighGreyLevelEmphasis derived from DWI, representing large uniform zones of high grey levels in the image. Previous studies have reported lower ADC values in carcinoma and high-risk tumors than in low-risk tumors [9,15-17]. Consistent with our findings, these previous studies defined the region of interest to exclude areas suggestive of necrosis and degeneration. The GLSZM_LargeZoneHighGreyLevelEmphasis, which showed significant differences relative to the LRG in our study, reflects high-signal areas in the DWI and corroborates previous findings. As reported by Abdel Razek et al., carcinoma and high-risk tumors exhibit histological features such as nuclear enlargement, hyperchromasia, nuclear contour keratinization, and cellular hyperplasia, which reduce the diffusion space of the extracellular matrix and intracellular and extracellular water protons, resulting in decreased ADC values [9].

Differentiation between LRG and HRG was effectively achieved with DWI, particularly using the coefficient of variation. However, previous studies have indicated that distinguishing between LRG and HRG is challenging. In this study, the AUC was 0.785, the lowest among the differentiations between WHO grades. Although CT is considered less effective for differentiating between LRG and HRG, MRI has demonstrated effectiveness, as LRG exhibits uniform signal characteristics, whereas HRG tends to show greater heterogeneity [18]. Additionally, necrosis and fat infiltration have been reported as features suggestive of high-risk tumors [19]. Furthermore, multiple studies indicate that the ADC of HRG is significantly lower than that of LRG [9,15,16], although case bias may influence this observation. Previous studies [9,15] have revealed a high overlap between WHO types B1 and B2. The signal variation in DWI is presumed to be effective in differentiating low-risk and high-risk tumors.

Among the evaluated features, morphological texture features extracted from T1-weighted images demonstrated the highest diagnostic performance for differentiating low-risk thymoma from thymic carcinoma (AUC = 0.933). These features characterize the geometric distribution of image intensities within the tumor and may provide complementary information beyond conventional visual assessment by reflecting intratumoral structural heterogeneity. The differentiation between low-risk thymoma and thymic carcinoma was effectively achieved using the position of high signal intensity on T1-weighted images. Although previous studies have reported significantly lower T1 values in invasive thymic epithelial tumors according to the Masaoka-Koga classification, no significant differences have been demonstrated according to the WHO classification [20]. Generally, low-risk thymomas are often non-invasive, whereas thymic carcinomas are more frequently invasive [5], which may partly explain the observed differences. Furthermore, hemorrhage and degenerative changes tend to occur more centrally in thymic carcinoma, which may influence the morphological texture features identified in the present study.

Unlike conventional studies that evaluate mean ADC values, the present study analyzed texture features extracted from ADC maps, enabling quantitative assessment of spatial heterogeneity within tumors rather than overall diffusivity alone. The differentiation between high-risk tumors and carcinoma using the ADC map was superior with the following four signal intensity-based indicators, demonstrating an AUC of 0.867: Intensity Variance (INTENSITY-BASED_IntensityVariance), Standard Deviation (INTENSITY-BASED_StandardDeviation), Maximum Intensity (INTENSITY-BASED_MaximumIntensity), and Intensity Interquartile Range (INTENSITY-BASED_IntensityInterquartileRange). These indicators are all related to signal variability. Previous MRI reports have indicated a high frequency of internal heterogeneity, supporting these findings [18].

An additional potential clinical advantage of MRI texture analysis is its ability to evaluate the entire tumor volume noninvasively. Image-guided biopsy samples only a limited portion of a tumor and may therefore be affected by sampling error, particularly in heterogeneous thymic epithelial tumors containing areas with different histological grades. In contrast, texture analysis incorporates information from the whole tumor volume and may better reflect intratumoral heterogeneity, thereby providing complementary information for preoperative assessment and reducing the risk of underestimating tumor aggressiveness.

Overall, our findings are generally consistent with previous MRI studies demonstrating the value of diffusion-weighted imaging and ADC-derived imaging biomarkers for differentiating thymic epithelial tumor subtypes [9,15]. Li et al. further reported that combining conventional ADC values with texture parameters improved diagnostic performance [12]. In contrast, the present study systematically evaluated texture features extracted independently from multiple MRI sequences, including T1-weighted images, T2-weighted images, diffusion-weighted images, and ADC maps. This approach enabled identification of the sequence-specific texture features with the highest diagnostic performance for each comparison, suggesting that different MRI sequences provide complementary information regarding intratumoral heterogeneity and may contribute to improved preoperative risk stratification of thymic epithelial tumors.

This study has some limitations. First, the sample size was small. Thymoma is a rare disease, and even prior studies have often been constrained by small sample sizes, a limitation also observed in this study. Although compiling cases from other institutions is necessary, establishing standardized imaging protocols for texture analysis is crucial. Second, there was a bias in the histological subtypes within LRG and HRG. Only one type A case was included in the LRG, while two-thirds of the HRG were type B2. Given the substantial overlap between type B2 and LRG observed in previous studies, the present study provides valuable insight into their potential differentiation. Conversely, the small number of type A cases, which are characterized by a high stromal component, may have influenced the results. However, type A is generally considered rare in Asia [5], and the distribution in this study is consistent with the regional distribution in Asia, potentially serving as an indicator for this population. Third, all VOIs were manually delineated by a single experienced radiologist. Although this approach ensured consistency throughout the study, intra-observer and inter-observer reproducibility were not evaluated and should be investigated in future studies. Furthermore, multicenter studies with larger cohorts are warranted to validate the present findings and improve their generalizability. In addition, further refinement and standardization of MRI texture analysis methods may improve their robustness and facilitate broader clinical application. Finally, this retrospective study did not include longitudinal follow-up data. Future prospective multicenter studies with larger cohorts and longitudinal follow-up are warranted to validate the reproducibility, generalizability, and clinical utility of MRI texture analysis for thymic epithelial tumors.

## Conclusions

MRI texture analysis demonstrated varying diagnostic strengths across MRI sequences and tumor subtypes. DWI-derived texture features were most useful for differentiating low-risk from high-risk thymoma, whereas T1-weighted morphological features showed the highest diagnostic performance for distinguishing low-risk thymoma from thymic carcinoma (AUC 0.933). Texture features extracted from ADC maps most effectively differentiated high-risk thymoma from thymic carcinoma (AUC 0.867). These findings support the complementary value of multiparametric MRI texture analysis for the preoperative WHO classification of thymic epithelial tumors. Nevertheless, these findings should be interpreted with caution because of the relatively small sample size and the lack of external validation.

## Appendices

**Table 2:** Top three texture features in diagnostic performance among pathological differences LRG: Low-risk thymoma group, HRG: High-risk thymoma group, TC: Thymic carcinoma, ADC: Apparent diffusion coefficient.

Feature	Label	Group1	Group2	Group_Comparison	AUC	CI_Lower	CI_Mid	CI_Upper
INTENSITY-BASED_IntensityBasedCoefficientOfVariation(IBSI:7TET)[]	b800	LRG	HRG	LRG vs HRGI	0.78535354	0.63345085	0.78535354	0.93725622
INTENSITY-BASED_IntensityBasedMeanAbsoluteDeviation(IBSI:4FUA)[]	ADC	LRG	HRG	LRG vs HRGI	0.77731092	0.61166059	0.77731092	0.94296126
INTENSITY-BASED_IntensityBasedMedianAbsoluteDeviation(IBSI:N72L)[]	ADC	LRG	HRG	LRG vs HRGI	0.77731092	0.61214944	0.77731092	0.94247241
MORPHOLOGICAL_RadiusRoiNorm-MaxIntensityCoor-PerimeterCoor-3DSmallestDist(IBSI:No)[]	in	LRG	TC	LRG vs TC	0.93265993	0.85802537	0.93265993	1
MORPHOLOGICAL_RadiusSphereNorm-MaxIntensityCoor-PerimeterCoor-3DSmallestDist(IBSI:No)[]	in	LRG	TC	LRG vs TC	0.91582492	0.82839526	0.91582492	1
MORPHOLOGICAL_RadiusRoiNorm-MaxIntensityCoor-PerimeterCoor-2DSagittalSmallestDist(IBSI:No)[]	in	LRG	TC	LRG vs TC	0.85858586	0.74138724	0.85858586	0.97578448
INTENSITY-BASED_IntensityInterquartileRange(IBSI:SALO)[]	ADC	HRG	TC	HRG vs TC	0.86734694	0.69955894	0.86734694	1
INTENSITY-BASED_IntensityVariance(IBSI:ECT3)[]	ADC	HRG	TC	HRG vs TC	0.86734694	0.71269549	0.86734694	1
INTENSITY-BASED_MaximumIntensity(IBSI:84IY)[]	ADC	HRG	TC	HRG vs TC	0.86734694	0.68226547	0.86734694	1
